# Vaccine rate forecast for COVID-19 in Africa using hybrid forecasting models

**DOI:** 10.4314/ahs.v23i1.11

**Published:** 2023-03

**Authors:** S Dhamodharavadhani, R Rathipriya

**Affiliations:** Department of Computer Science, Periyar University, Salem-India

**Keywords:** Vaccination forecasting, ARIMA, Immunization, Time series techniques, *Hybrid ARIMA*, *Prediction*, *Linear Regression*, *Gaussian Regression Process*, *Hybrid GRNN*

## Abstract

**Background:**

The public health sectors can use the forecasting applications to determine vaccine stock requirements to avoid excess or shortage stock. This prediction will ensure that immunization protection for COVID- 19 is well-distributed among African citizens.

**Objective:**

The aim of this study is to forecast vaccination rate for COVID-19 in Africa

**Methods:**

The method used to estimate predictions is the hybrid forecasting models which predicts the COVID-19 vaccination rate (CVR). HARIMA is a hybrid of ARIMA and the Linear Regression model and HGRNN is a hybrid of Generalized Regression Neural Network (GRNN) and the Gaussian Process Regression (GPR) model which are used to improve predictive accuracy.

**Results:**

In this study, standard and hybrid forecasting models are used to evaluate new COVID-19 vaccine cases daily in May and June 2021. To evaluate the effectiveness of the models, the COVID-19 vaccine dataset for Africa was used, which included new vaccine cases daily from 13 January 2021 to 16 May 2021. Root Mean Squared Error (RMSE) and Error Percentage (EP) are used as evaluation measures in this process. The results obtained showed that the hybrid GRNN model performed better than the hybrid ARIMA model.

**Conclusion:**

HGRNN model provides accurate daily vaccinated case forecast, which helps to maintain optimal vaccine stock to avoid vaccine wastage and save many lives.

## Introduction

**Figure 1:**
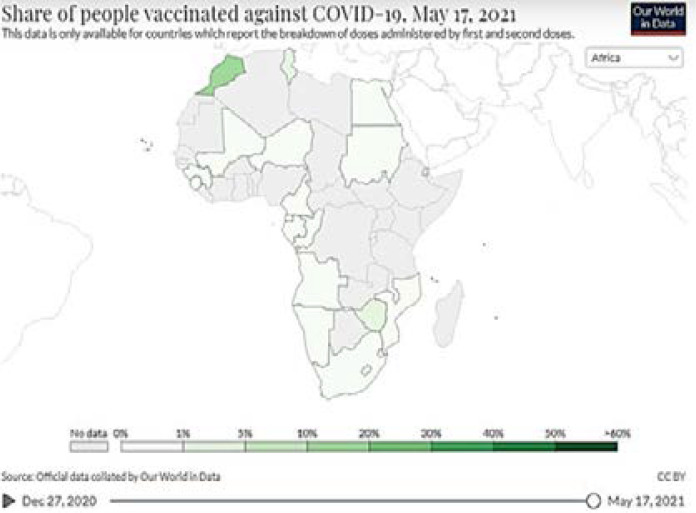
Number of People Vaccinated in Africa

Worldwide, COVID-19 vaccination is one of the greatest supply and logistics challenges facing us to date. In the current context of the COVID-19 epidemic, many countries are facing vaccine shortages. The COVID-19 vaccine dose has been administered 950 million times worldwide so far, at approximately 12 doses per 100 people. However, in countries such as United States, Israel, China, and Russia, more than 50% of people are vaccinated against COVID-19. Conversely, many countries in Africa have not yet begun the vaccination process in full swing and their vaccination rate is less than 1%. Their frontline workers also have not been vaccinated so far. Figure 1 shows the breakdown of COVID-19 vaccination by those who have been partially or completely vaccinated in Africa. Some believe that machine learning and block chain technology can help with this task 1–4.

One of the key factors for effective supply chain management is the forecasting of health products. COVID-19 has caused significant and unprecedented pressure in most of the products' supply chains worldwide. Previous research work related to the forecasting of vaccine supply chain disruption identified some factors that lead to it 1–6. The early prediction of excess demand of vaccine during epidemics will have significant implications for supply chain managers and government officials.

The current epidemic and global health crisis have worsened health supply chains and caused significant disruptions in upstream and downstream operations. Severe shortages, logistic challenges and travel restrictions have also added fuel to it^7^–^8^. Because of these constraints, accurate short-term forecasts have become an important decision-making tool and are essential to the medium and long-term forecasting supply chain processes. Therefore, it is necessary and useful to predict the vaccination rate in the coming days to handle vaccine stocks effectively against wastage and supply chain disruption. Under this scenario, this study explores how a hybrid forecasting model can be used for accurate short-term forecasting of vaccination rates.

Auto Regressive Integrated Moving Average (ARIMA) model is also called as Box- Jenkins (BJ) method 9. ARIMA models 10 have been widely used for detecting outbreaks of infectious diseases 11, 12, 13, 14. The stationarity of the time series is essential for a well fitted ARIMA model. A detailed study of this model showed that time series data with sparse data could not be modeled well 15. The ARIMA model is often used in the field of infectious diseases to predict its trajectory. Also used to model the relationship between rainfall and temperature and the disease 16 and the study relationship between suicide cases and national alcohol policies 17. Several researchers proposed modeling of COVID-19 using time series techniques 18–26. Zhang 27 provided a hybrid predictive model that included both ARIMA and ANN. Ju et al. 28 developed a hybrid forecasting model that used the moving average approach by integrating a modified particle swarm optimization (MPSO) method to improve computational performance. ARIMA model is also combined with Non-linear Autoregressive (NAR) neural network to enhance their forecasting accuracy 29.

A combination of GPR and adaptive neuro-fuzzy inference system (ANFIS) 10 used in groundwater level forecasting. In 30, an extensive comparative study was carried out between several surrogate models, comprising GPR, using simulation-optimization methodology with uncertainty parameters. In the end, they had concluded that the GPR models and their ensemble were efficient methods concerning prediction accuracy. GRNN model was built in as a new computational method for the field of incidence prediction of infectious diseases. Authors of 31 developed a GRNN network with a one-dimensional input and output layer to predict blood, and sexually transmitted infections are occurring. In 32, authors implemented a comparison analysis on Back Propagation Neural Network (BPNN), GRNN, and RBFNN network for prediction of the evaporation. The results showed that the GPR is a successful technique compared with artificial neural network approaches. However, very few works related to the vaccine prediction system using ARIMA and neural network have been proposed in the literature 33, 34. In 35, authors have used ARIMA model to forecast the total number of fully vaccinated people against COVID-19 in the Asia, United States, Africa, Europe, South America, etc.

In the literature, there are numerous studies on the prediction of COVID-19 cases and/or mortality using the ARIMA model. This demonstrates the effectiveness of the ARIMA model but still has the potential to improve the ARIMA model, which reduces the prediction error by hybridizing with the error reduction model. Nevertheless, there is a gap in the literature on estimating the usage trend of COVID-19 vaccines by using a variant of the ARIMA time series model.

The main contributions of this research work are as follows:

1. To forecast COVID-19 Vaccine Rate for Africa using Hybrid Forecasting Models such as Hybrid ARIMA and Hybrid GRNN

2. Linear Regression model and Gaussian Process Regression model are used as Error or residual forecasting model

3. Root- Mean-Squared Error (RMSE) is used as performance indicator to assess the standard and hybrid version of forecasting models.

4. Identify the most appropriate predictive method for COVID-19 Vaccine dataset.

Use the best model for future forecasting of CVR for Africa

This research paper presents a hybrid forecasting methodology to develop a highly accurate COVID-19 vaccination rate prediction model for Africa. HARIMA is the combination of ARIMA and Linear Regression model which is used to improve the predictive accuracy. The time series Covid-19 vaccination dataset from https://ourworldindata.org/coronavirus-source-data is used to predict the vaccination rate for Africa. These results will be used by health professionals and government officials to plan vaccination strategies that could avert the coming COVID-19 epidemic in the country and save millions of people from this deadly disease.

## Methodology Methods

### Design Goals

The primary design goals of the hybrid forecasting model are:

Generating an accurate forecasting of vaccination rate for Africa using time-series vaccination data.

Reduce the prediction error by introducing the error forecasting model with the ARIMA and GRNN models.

### Hybrid ARIMA Forecasting Model

Generally, predictions based on historical time-series epidemiological data do not always produce accurate future predictions. This is because these predictive models used relationships derived from historical data to predict the future by implicitly assuming that there are certain trends in the dataset. Similarly, the predictions of the ARIMA model have the residuals in their forecasting. Therefore, to provide a more accurate forecast model, the residual or error forecast model is integrated with the ARIMA model.

The main aim of the hybridization is to provide the higher prediction accuracy models for epidemiological data. Linear Regression (LR) model is used to forecast the residual of ARIMA. LR models finds the linear relationship between the predicted and actual residual values. It is mathematically represented as in equation 1.

YR= ψ0 + ψ1 XRi +Ei     (1)

Were

YR-Predicted residual values

ψ0 - Intercept

ψ1 – Slope Co-efficient

XRi - residual values of SI based Statistical Predictive Models Ei - Random error term

The incorporation of trends from the residuals of the ARIMA model into the Linear Regression forecast model will result in higher accuracy. The steps in algorithm-1 are used to combine the ARIMA model with Linear Regression forecast mode

**Figure:**
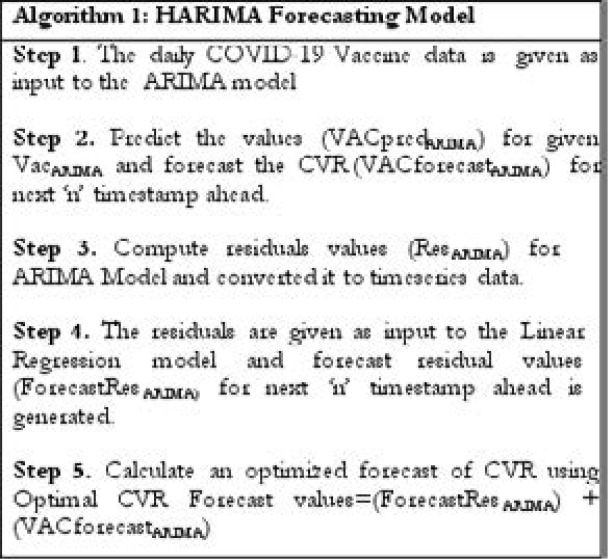


### Hybrid GRNN (HGRNN) Forecasting Model

Gaussian process is a machine learning technique 31]used to make uncertain predictions. It's also described as a finite discrete random variable collectively transferred in the Gaussians 30. These random variables represent the value for a function f(x) at input x in regression problems. It is denoted as {f(x): x X}, mean function µ(x) and the covariance function k (x, ∈ j) therefore it can be shown as in equation (2)

*f (·) ∼ GP (µ (·), k (·,·))*     (2)

A covariance function as defined in equation (3) is used to represent the covariance between pairs of random variables in GP.



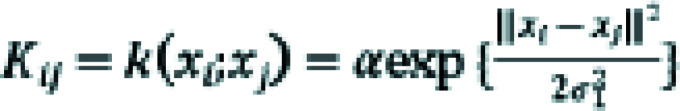



Where σ1= Characteristic length scale, α = Signal variance

A special case of Radial Basis Networks (RBN) is the Generalized Regression Neural Network (GRNN)^32^. The configuration of GRNN with two layers remains comparatively simple besides fixed. The first is the sequence, and the second is a summation. When the input is passed over every cmponent in the pattern layer, the input-response association will stand “memorized” and stored within the component. Consequently, the number of components in the pattern layer is equivalent to the total of individual values in the training set. In each pattern unit, a Gaussian PDF is applied to the network input, so that it is defined as equation 4.



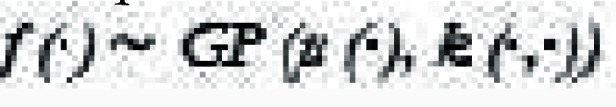



where Θ is the output of the Pattern Unit, A is the origin, t is the vector of training stored in the unit, and σ is a positive variable known as the “distance” or “smooth parameter” or “smoothing factor”. If Θ is determined, the calculation is transferred to the summation layer P = SUM (P * Θ) / SUM (Θ) where P is the conditional prediction of P and Q is the solution in the sample of training. The steps in algorithm-2 is used to combine the GRNN model with GPR error forecast model.

**Figure:**
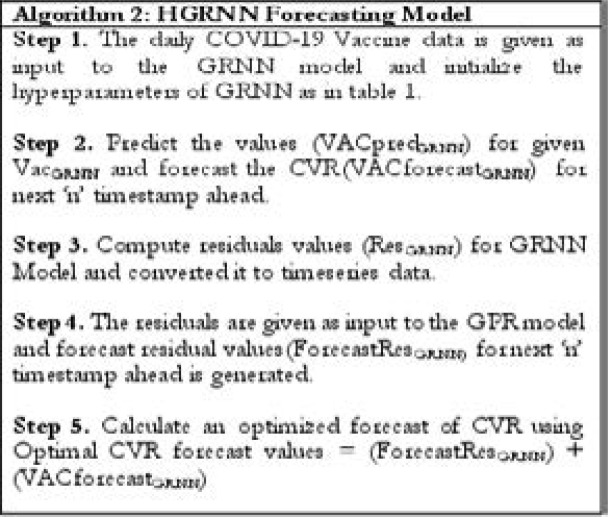


### Evaluation Metrics

The followings metrics as shown in equations 5 and 6 are used as an experimental metrics to assess the performance of the proposed methodology 37–39.

*RMSE* = (mean ((t ― y). ^2));     (5)

*PE* = (ab (y― t)/sum(t) * 100;     (6)

Where t-actual data y-target data, sum(.)- is the summation of the values in the vector, abs(.)- is the absolute value.

A non-seasonal ARIMA model is referred to as ARIMA (p, d, q), where p is the number of auto-regressive terms, d is the number of variations required to make the time series a stationary, and q is the number of moving average term. Akaike's Information Criterion (AIC) can be used to identify the best value for p,q, and d for ARIMA model . Augmented Dickey- Fuller (ADF) test is used in this work to find the optimal value of differencing. Initially, load te COVID-19 Vaccination time series dataset for Africa into the MATLAB toolbox environment then trained the ARIMA forecasting model with the optimal values of hyper parameter values (p, q, d). After that, predict the values of CVR using tested model and check model's accuracy using metrics given in eq. 5 and eq.6.

## Experimental Results

This section assesses the performance of the proposed methodology by comparing the standard ARIMA, Hybrid ARIMA, standard GRNN and Hybrid GRNN models for CVR prediction. To analyse the accuracy of the above forecasting models, experimental metric like Root Mean Square Error (RMSE) and Percentage of Error (PE) are used. Dataset is extracted from the archive of https://ourworldindata.org/[36] which contains cumulative vaccination count from 13.01.2021 to 16.05.2021 for Africa.

Table 1 shows the optimal value of hyperparameters which are used in GRNN and ARIMA forecasting models.

**Table 1:** List of Hyperparameter

Hyperparameters	Values
Activation Function	Hyperbolic tangent sigmoid
Training Algorithm	Bayesian Regularization
Number of neurons in Hidden Layer	25
Number of Feedback Delay	4
Spread	0.5
ARIMA(p,q,d)	(7,1,2)

**Figure 2:**
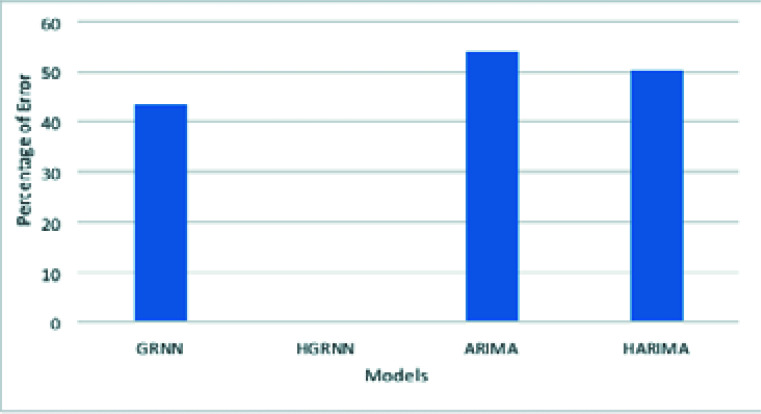
Comparative Performance of Forecasting models

Tables 2 shows the RMSE and PE values of the ARIMA, Hybrid ARIMA, GRNN and HGRNN models respectively. Figure 2 shows a bar chart of the PE value of all forecasting models. The hybrid GRNN model has a much lower percentage of error than all other models.

**Table 2:** Performance of Forecasting Models

Models	RMSE	PE
GRNN	136288	43.47292
HGRNN	859.09	0.42205
ARIMA	186917.7	54.21174
HARIMA	149746	50.26698

**Figure 3:**
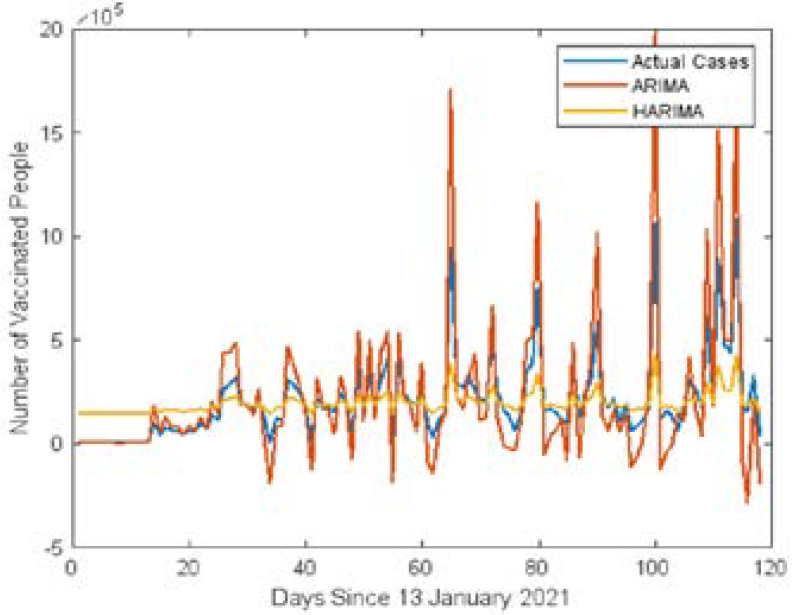
Comparison of Predicted cases of ARIMA and Hybrid ARIMA

**Figure 4:**
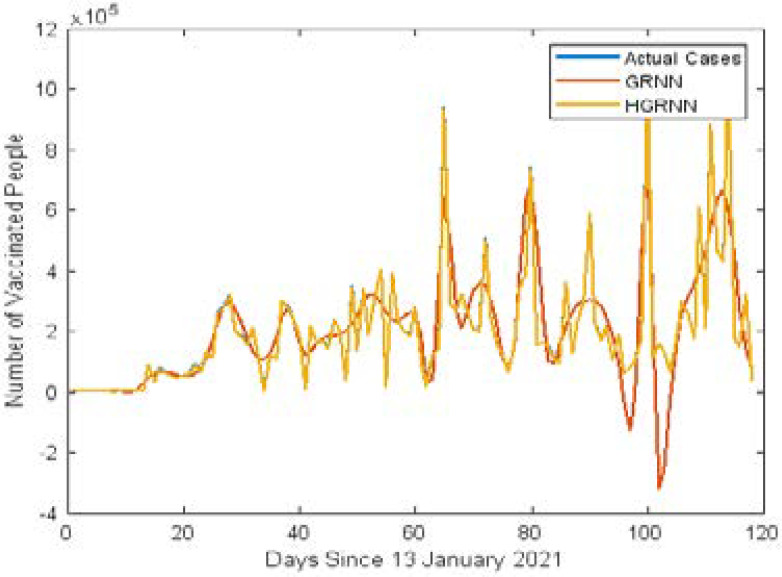
Comparison of Predicted cases of GRNN and Hybrid GRNN

Figure 3 shows the line plot of actual and predicted vaccinated cases using ARIMA and Hybrid ARIMA models. Figure 4 shows the line plots of actual and predicted vaccinated cases using GRNN and Hybrid GRNN models.

## Discussions

In Tables 2 shows the RMSE and PE values of the ARIMA, Hybrid ARIMA, GRNN and HGRNN models respectively. The RMSE value of the hybrid ARIMA model is 149746, which shows that the hybrid model enhances the accuracy of the predicted value of COVID-19 vaccination cases by 19.89%. Similarly, the RMSE value of the hybrid GRNN model is 859.09, which shows that the hybrid GRNN model enhances the accuracy of the predicted value of vaccinated cases by 99.37% over the standard GRNN model. On the other hand, the hybrid GRNN model increases the accuracy of the predicted vaccinated cases by 99.42% over the hybrid ARIMA model. Therefore, it has been decided that hybrid GRNN is the best forecasting model for CVR in Africa.

The hybrid GRNN model accurately captures the trend of the vaccination dataset, so there is no visible blue line plot on the chart.

**Figure 5:**
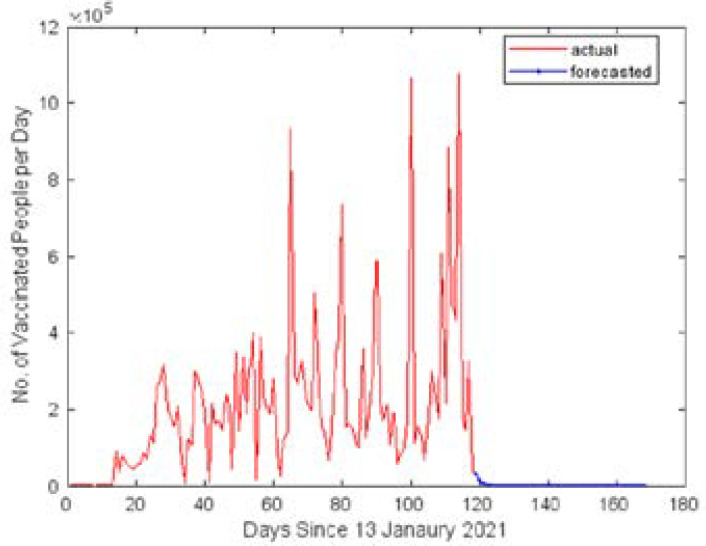
Forecasted Vaccinated Cases for Africa

Figure 5 shows the line plot for the predicted COVID-19 vaccine cases for the period from May 17 to July 5, 2021. It is clear from the forecast that the number of vaccinated cases per day may stagnate after June 2021. This indicates that the existing vaccination stock in Africa may not be sufficient to distribute the vaccine on a large scale. This is may be due to the global vaccine shortage and poor supply chain management. Based on the results of the forecasting model and the current status of COVID-19, a rapid increase in the number of vaccinated cases is expected to control the spread and reduce the mortality rate. The next 50 days forecasted vaccinated cases per Day and CVR in Africa using hybrid GRNN model are clearly shown in Table 3. The forecasted CVR is very poor for Africa so government officials should take necessary steps to increase the vaccination process in the country.

**Table 3:** Forecasted COVID-19 Vaccinate Rate (CVR) for Africa

Date	Forecasted Vaccinated cases per day	COVID-19 Vaccinate Rate	Date	Forecasted vaccinated cases per day	COVID-19 Vaccinate Rate
17-May-21	32215	0.073	11-Jun-21	1031	0.002
18-May-21	14613	0.033	12-Jun-21	1031	0.002
19-May-21	6729	0.015	13-Jun-21	1031	0.002
20-May-21	3321	0.008	14-Jun-21	1031	0.002
21-May-21	1898	0.004	15-Jun-21	1031	0.002
22-May-21	1327	0.003	16-Jun-21	1031	0.002
23-May-21	1112	0.003	17-Jun-21	1031	0.002
24-May-21	1038	0.002	18-Jun-21	1031	0.002
25-May-21	1019	0.002	19-Jun-21	1031	0.002
26-May-21	1017	0.002	20-Jun-21	1031	0.002
27-May-21	1020	0.002	21-Jun-21	1031	0.002
28-May-21	1024	0.002	22-Jun-21	1031	0.002
29-May-21	1026	0.002	23-Jun-21	1031	0.002
30-May-21	1028	0.002	24-Jun-21	1031	0.002
31-May-21	1029	0.002	25-Jun-21	1031	0.002
01-Jun-21	1030	0.002	26-Jun-21	1031	0.002
02-Jun-21	1031	0.002	27-Jun-21	1031	0.002
03-Jun-21	1031	0.002	28-Jun-21	1031	0.002
04-Jun-21	1031	0.002	29-Jun-21	1031	0.002
05-Jun-21	1031	0.002	30-Jun-21	1031	0.002
06-Jun-21	1031	0.002	01-Jul-21	1031	0.002
07-Jun-21	1031	0.002	02-Jul-21	1031	0.002
08-Jun-21	1031	0.002	03-Jul-21	1031	0.002
09-Jun-21	1031	0.002	04-Jul-21	1031	0.002
10-Jun-21	1031	0.002	05-Jul-21	1031	0.002

## Recommendations to Health Officials

1. Public health programmes should focus on increasing awareness of the benefits of the COVID-19 vaccine and reducing the perceived adverse effect.

2. Showcase the medical proof for the safety and efficiency of COVID-19 vaccines which are key factors to improve the CVR in Africa.

3. Promoting COVID-19 vaccination awareness campaign

4. Increase the vaccine stock and reduce the inequalities in access to COVID-19 vaccines due to financial restrictions.

However, no research has used statistical and machine learning predictions on region-level COVID-19 vaccines using both infodemiological web information and clinical data. As a result, we concentrated on developing more precise methods for forecasting COVID-19 vaccination rates by combining clinical and web data.

## Conclusion

In this study, standard and hybrid forecasting models are used to evaluate new COVID-19 vaccine cases daily in May and June 2021. To evaluate the effectiveness of the models, the COVID-19 vaccine dataset for Africa was used, which included new vaccine cases daily from 13 January 2021 to 16 May 2021. RMSE and error percentage are used as evaluation measures in this process. The results obtained showed that the hybrid GRNN model performed better than the hybrid ARIMA model. This prediction of new vaccine cases is made with current time series data for Africa. However, this can be improved by taking some awareness measures and increasing the availability of vaccines by the public health departments. In the future, a new methodology using optimization algorithms will be proposed to fix the hyper-parameters of the GRNN model for forecasting the COVID-19 vaccinated cases. Another future goal is to predict the number of people who will be fully vaccinated for an area with deep learning time-series models. A performance study will be conducted to identify the cost-effective method.
